# Fluorescent sensing copolymers: Synthesis, nanofiber fabrication and application in picric acid sensors

**DOI:** 10.1016/j.heliyon.2024.e40786

**Published:** 2024-11-29

**Authors:** Hong-Jia Lin, Po-Hsiang Tseng, Wen-Chung Wu

**Affiliations:** Department of Chemical Engineering, National Cheng Kung University, Tainan, 702, Taiwan

**Keywords:** Fluorescent sensor, Electrospinning, Nanofibers, Picric acid

## Abstract

Increasing attention has been paid to the detection of explosives due to the occurrence of terrorist attacks around the world. Here, we used free radical polymerization to develop two different types of fluorescent copolymers for use in detecting picric acid. One exhibits aggregation-caused quenching (ACQ) and is called PNNS [poly (*N*-isopropyl acrylamide-*co*-*N*-hydroxymethyl acrylamide -*co*-styrene-pyrene), poly (NIPAAm-*co*-NMA-*co*-St-Py)]. The other possesses aggregation-induced emission (AIE) properties and is called PNNP [poly (*N*-isopropyl acrylamide-*co*-*N*-hydroxymethyl acrylamide-*co*-2-(1,2,3,4,5-pentaphenyl-1H-silol-1-yloxy) ethyl methacrylate), poly (NIPAAm-*co*-NMA-*co*-PPS-HEMA)]. Nanofibrous thin films of these copolymers were obtained by electrospinning. Upon interaction with picric acid, the fluorescence intensity of each copolymer was quenched due to photo-induced electron transfer (PET). The average diameters of PNNS and PNNP nanofibers were 179 ± 28 nm and 235 ± 143 nm, respectively. Sensing performance was evaluated by Stern-Volmer analysis. The Stern-Volmer constant (K_sv_) values for PNNS and PNNP nanofibers were 0.012 μΜ^−1^ and 0.119 μΜ^−1^, respectively. Since the aggregated state of PNNP nanofibrous thin films can increase dramatically, the AIE property of this material provides a large dynamic range. Finally, the reusability of water- and methanol-washed nanofiber thin films was tested, revealing that the nanofiber sensors were reusable for detecting picric acid.

## 1 Introduction

Over the past few decades, the detection of explosive compounds such as picric acid has become an increasingly important concern around the world due to terrorist attacks. Many methods have been developed and applied to detect explosives, including spectroscopy (mass, infrared, Raman spectroscopy) [1,2], animal sniffing [3], and sensors (chemical, electrochemical, fluorescence) [4,5]. Among these methods, fluorescent sensors are especially attractive due to their high selectivity, good sensitivity, and ease of application [6,7].

Fluorescence is a molecular property that involves light-matter interactions. After a molecule absorbs energy from an incident photon, its electronic state may be promoted from a ground state to an excited state. Fluorescence will occur if the excited molecule returns to its ground state via radiative release of the excitation energy. However, the fluorescent emission may be diminished if excited molecules are in close proximity to quenching molecules that can absorb the excitation energy. Such quenching interactions may be mediated by photoinduced electron transfer (PET) and energy transfer. This physical phenomenon of fluorescence quenching can be exploited to develop probes for detecting explosive compounds. For instance, Namgung and colleagues leveraged PET interactions to develop a tetraphenylethylene-conjugated microporous polymer (TPE-CMP)-based sensor for picric acid that exhibited better sensitivity than a standard TPE-containing linear polymer (TPE-Ph) due to its 3D porous structure [8]. Using a different approach, Kumar et al. designed a Fӧrster Resonance Energy Transfer (FRET)-based sensor system for detecting explosives. In this system, poly-tryptophan and carbazole comprise a FRET pair with tryptophan acting as a donor and carbazole as an acceptor. This electron-rich FRET pair was used to detect nitroaromatic compounds through PET, which causes a change in FRET efficiency. Importantly, the fluorescence properties of the system were not perturbed by environmental conditions due to the unique characteristics of this FRET strategy [9].

In general, fluorescent molecules can be divided into two categories depending on their emission behavior. One category comprises molecules subject to an aggregation-caused quench (ACQ) effect, while molecules in the other category undergo aggregation-induced emission (AIE). For molecules with the AIE property, fluorescence increases upon the occurrence of aggregation due to the restriction of intramolecular motion (vibration or rotation) [10,11]. In contrast, molecules that exhibit fluorescence decreases due to aggregation possess the ACQ property [12]. With regard to fluorescent probes, molecules with AIE characteristics are widely used in explosive detection applications due to their intense fluorescence upon aggregation [13,14]. For example, Zhou and colleagues used TPE-containing polymers (with AIE properties) to detect nitro-compounds through electron transfer [15].

Electrospinning is a well-known technique for generating nanofibers with higher surface-to-volume ratios than are achievable with micron-sized fibers or film types. In the electrospinning process, a polymer solution or melt is placed into a syringe with an attached metal needle. As the polymer solution is extruded through the needle, a high voltage is applied to exert an electrostatic repulsion force on the polymer. If this force is greater than the surface tension of the polymer drops, the drops will be whipped into a collector and form a nanofibrous film. Several polymer properties are known to influence the morphology of resultant nanofibers, such as solution viscosity, dispersity index and electrical conductivity [[16], [17], [18]]. An essential step for developing a successful electrospinning process is optimization of process control parameters to create a desired product. Due to the high surface-to-volume ratios of electrospun nanofibers, these materials are widely used in many applications [[19], [20], [21]].

In this work, two fluorescent monomers, styrene-pyrene (St-Py) and 2-(1,2,3,4,5-pentaphenyl-1H-silol-1-yloxy) ethyl methacrylate (PPS-HEMA) were prepared according to our previous reports [22,23] and used to react with two non-fluorescent monomers, NIPAAm and NMA. The mixtures were used to synthesize two series of random PNNS or PNNP copolymers. PNIPAAm is a temperature-sensitive polymer; when the temperature rises above a lower critical solution temperature (LCST), the material will become hydrophobic [24]. NMA can form crosslinks when heated, which prevents the copolymer from dissolving in water or other solvent environments [25]. Pyrene is an fluorescent molecule with ACQ properties that is widely used to construct fluorescent sensors due to its desirable fluorescence properties of long fluorescence lifetime, high quantum yield, and ability to form excimers [26]. Pyrene has been used widely to detect nitroaromatic (NA) explosive compounds. In this application, the electron-rich pyrene binds to electron-deficient explosives due to π-π interactions, which facilitates PET-mediated fluorescence quenching [[27], [28], [29]]. In our previous study, we used PPS-HEMA (with AIE characteristics) as an indicator for micelle encapsulation [22], but in this work, PPE-HEMA was used to detect electron-rich picric acid. With these materials, solid fluorescent probes were synthesized by electrospinning. We chose to develop solid-state sensors due to their advantages over solution-state sensors, including portability, operational simplicity and potential reusability.

## 2 Materials and methods

### 2.1 Synthesis of PNNS

#### 2.1.1 Materials

4-Bomostyrene (Sigma-Aldrich), pyrene-1-boronic acid (Sigma-Aldrich), potassium phosphate (K_3_PO_4_, from J.T.Baker), azobisisobutyronitrile (AIBN, UniRegion), tetrakis(triphenylphosphine)palladium(0) (Pd(pph_3_)_4_, Sigma-Aldrich), magnesium sulfate anhydrous (MgSO_4_, SHOWA), *N*-isopropyl acrylamide (NIPAAm, Sigma-Aldrich), *N*-hydroxymethyl acrylamide (NMA, Sigma-Aldrich), ethyl acetate (EA), *n*-hexanes, methanol, toluene, and dimethyl sulfoxide (DMSO) were purchased and used as received.

#### 2.1.2 Synthesis of poly (NIPAAm-*co*-NMA-*co*-St-Py), PNNS (Scheme 1)

Azobisisobutyronitrile (6.00 mg, 0.037 mmol), NIPAAm (1.00 g, 8.80 mmol), NMA (447 mg, 4.4 mmol), and St-Py (0.09 g, 0.295 mmol) were placed in a flask under N_2_ atmosphere for 20 min. Then, MeOH/toluene (volume ratio = 1:1) was added to the flask and reacted at 65 °C for 1 day. Afterward, the reaction mixture was slowly dripped into diethyl ether/toluene (volume ratio = 10:1) to stimulate precipitation. The precipitates were filtered and dried in an oven, yielding a light-yellow product (1.02 g).

**Scheme 1:**
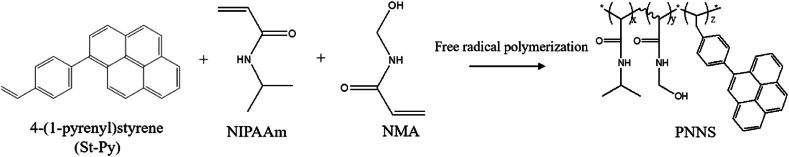
Synthesis of PNNS.

### 2.2 Synthesis of PNNP

#### 2.2.1 Materials

Diphenylacetylene (Sigma-Aldrich), lithium (Chemetal), triethylamine (Sigma-Aldrich), trichlorophenylsilane (Sigma-Aldrich), 2-hydroxyethyl methacrylate (HEMA, Sigma-Aldrich), benzyl triethylammonium chloride (Sigma-Aldrich), N-isopropyl acrylamide (NIPAAm, Sigma-Aldrich), N-hydroxymethyl acrylamide (NMA, Sigma-Aldrich), methanol, toluene, n-hexanes, diethyl ether, dimethyl sulfoxide (DMSO), and ethyl acetate (EA) were purchased and used as received.

#### 2.2.2 Synthesis of poly [NIPAAm-*co*-NMA-*co*-2-(1,2,3,4,5-pentaphenyl-1H-silol-1-yloxy) ethyl methacrylate], PNNP (Scheme 2)

Azobisisobutyronitrile (6.00 mg, 0.037 mmol), NIPAAm (1.00 g, 8.80 mmol), NMA (447 mg,4.4 mmol), and PPS-HEMA (250 mg, 0.423 mmol) were placed in a flask under N_2_ atmosphere for 20 min. Then, MeOH/toluene (volume ratio = 1:1) was added to the flask and reacted at 65 °C for 1 day. Afterward, the product was slowly dripped into diethyl ether/toluene (v/v = 10) to stimulate precipitation. The precipitate was filtered and dried in an oven, yielding a light-yellow product (1.00 g).

**Scheme 2:**
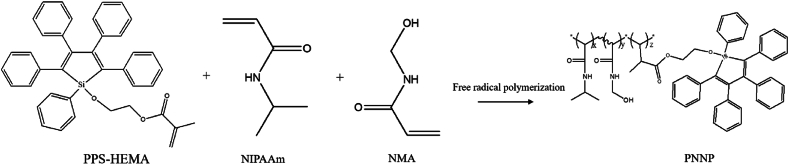
Synthesis of PNNP.

### 2.3 Preparation of electrospun nanofiber thin films

PNNS (13 wt%) or PNNP (16.67 wt%), and benzyl triethylammonium chloride (BTEAC, 5 wt% polymer) were dissolved in methanol and stirred for 1 day. The polymer solution was put into a glass syringe with a metal needle and used to prepare electrospun nanofibers at the parameters shown in Table 1.

**Table 1:** Electrospinning parameters for PNNS and PNNP.

	PNNS	PNNP
**Concentration (wt.%)**	13	16.67
**Electric voltage (kV)**	10	12
**Flow rate (ml/min)**	0.008	0.005
**Working distance (cm)**	15	15
**Collector**	aluminum sheet	aluminum sheet
**Collection time (h)**	4	12

### 2.4 Characterization of random copolymers

Fluorescent monomers or polymers were identified by NMR spectrum (Bruker Avance 600 NMR). The morphology of nanofibers was monitored by field emission scanning electron microscopy (FE-SEM) with a Hitachi-SU8010 at an acceleration voltage of 10 kV. Fifty randomly selected nanofibers were measured to obtain the average diameter.

A JASCO V-550 UV–vis spectrophotometer was used to analyze the weight percentage (wt.%) of fluorescence monomer in the copolymer according to a calibration curve. The calibration curves for fluorescent monomers were obtained as follows.

(1) St-Py calibration curve:

Different concentrations of St-Py solution were prepared in MeOH (2, 4, 6, 8, 10, 20, 40 μM), and absorption was measured at a wavelength of 344 nm.

(2) PPS-HEMA calibration curve:

Different concentrations of PPS-HEMA solution were prepared in MeOH (20, 40, 60, 80, 100, 200 μM), and absorption was measured at a wavelength of 376 nm.

### 2.5 Fluorescence quenching experiment

The fluorescence intensity of each copolymer was recorded in the presence of different concentrations of picric acid solution using a Hitachi F-2500 Fluorescence Spectrophotometer. The fluorescent spectra of PNNS were recorded from 350 to 600 nm, with an excitation wavelength of 344 nm. The fluorescent spectra of PNNP were recorded from 400 to 700 nm, with an excitation wavelength of 376 nm.

To compare the sensing abilities of different types of copolymers, both copolymers were prepared as a solution, nanofiber thin film and spin coating film, which were used for detection of picric acid.

(1) Solution type:

For PNNS, 3 ml of a solution (MeOH/water = 1/1, v/v) with PNNS (containing 2 μM Sy-Py monomer) was mixed with different concentrations of picric acid (500, 400, 300, 200, 100, 50, 40, 30, 20, 10, 0 μM).

For PNNP, 1 ml of a solution (THF/water = 1/4, v/v) with PNNP (containing 77 μM PPSHEMA monomer) was mixed with different concentrations of picric acid (25, 20, 15, 10, 5, 0 μM).

(2) Nanofiber:

For PNNS, a nanofiber thin film was fixed on a quartz plate with tape. The plate was inserted into a quartz cell with 3 ml mixed solution (MeOH/water = 1/1, v/v) containing different concentrations of picric acid (500, 400, 300, 200, 100, 50, 40, 30, 20, 10, 0 μM).

For PNNP, a nanofiber thin film was fixed on a quartz plate with tape. The plate was inserted into a quartz cell with 1.5 ml mixed solution (THF/water = 1/4, v/v) containing different concentrations of picric acid (25, 20, 15, 10, 5, 0 μM).

(3) Spin coating material:

For PNNS, PNNS solution (13 wt% in MeOH) was added dropwise onto a quartz plate and spun with the following parameters (2000 rpm for 30 s) to obtain a spin coating thin film. Then, the plate was inserted into a quartz cell with 3 ml mixed solution (MeOH/water = 1/1, v/v) containing different concentrations of picric acid (500, 400, 300, 200, 100, 50, 40, 30, 20, 10, 0 μM).

For PNNP, PNNS solution (9 wt% in MeOH) was added dropwise to a quartz plate and spun with the following parameters (2000 rpm for 30 s) to obtain a spin coating thin film. Then, the plate was inserted into a quartz cell with 1.5 ml mixed solution (THF/water = 1/4, v/v) containing different concentrations of picric acid (25, 20, 15, 10, 5, 0 μM).

### 2.6 Reusability of nanofibrous films

A random copolymer nanofibrous film was fixed on a quartz slide and inserted into a quartz cell with picric acid (500 μM for PNNS, and 100 μM for PNNS). Then, the fluorescence intensity was measured. After the measurement was made, MeOH and water were used to rinse the nanofibrous film. After the rinse, the nanofibrous film was used again to detect picric acid and evaluate its reusability.

## 3 Results and discussion

### 3.1 Synthesis of random copolymer

#### 3.1.1 Synthesis of PNNS

**Fig. 1:**
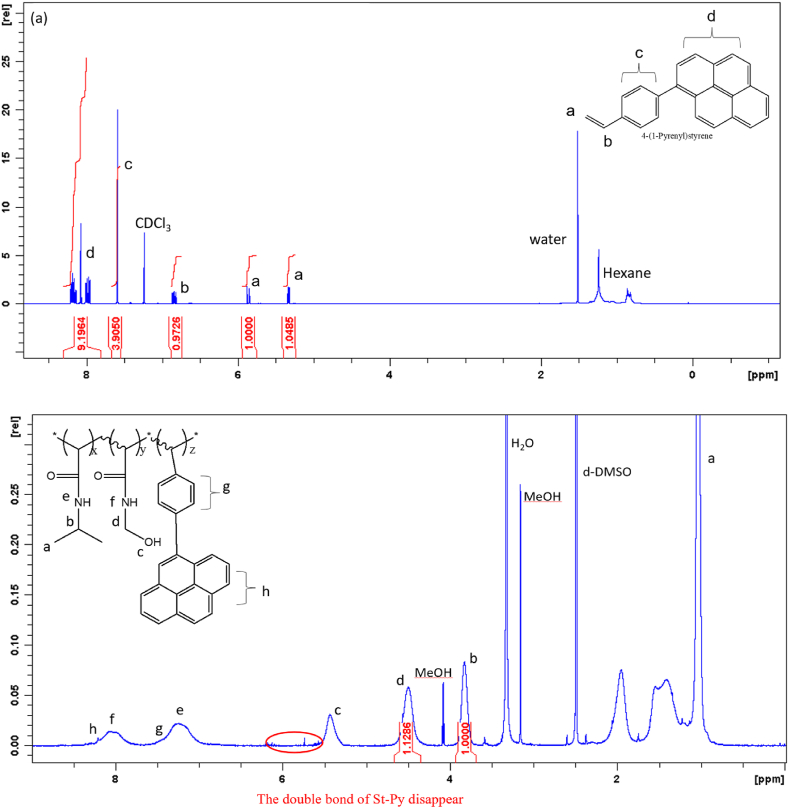
NMR spectra of (a) St-Py (b) PNNS.

Random copolymers [poly (NIPAAm-*co*-NMA-*co*-St-Py), PNNS] functionalized with pyrene moieties were prepared in a two-step synthesis procedure. In the first step, 4-(1-Pyrenel) styrene (St-Py) was synthesized via a Suzuki coupling reaction using pyrene-1-boronic acid and bromostyrene as reagents and Pd(PPh_3_)_4_ as a catalyst [29]. Then, the random copolymer which developed by our team was synthesized via a free radical polymerization reaction with St-Py, NIPAAm and NMA as monomers and AIBN as an initiator; the mixed solution was reacted at 65 °C for 1 day and then subjected to reprecipitation for purification. The structures of St-Py and PNNS were inspected by NMR (Fig. 1). The chemical shift of the double bond in St-Py (Fig. 1(a), δ: 5.4 and 5.9 ppm) disappeared after polymerization, indicating the reaction was successful. In addition, quantification of the proton peak areas shown in Fig. 1(b) (peaks “b” and “d”) allowed us to calculate the ratios of monomers (NMA and NIPAAm). The results showed that the ratio of peak “b” (NIPAAm) to peak “d” (NMA) in the PNNS NMR spectrum was 1.00:1.13. Since the proportion of fluorescent molecules was relatively low, the percentage of fluorescence monomer in PNNS was measured from the UV spectrum using a St-Py calibration curve (Fig. S1). The results showed that PNNS copolymer contained 6.37 wt% St-Py, which allowed us to quantify the concentration of fluorescent monomer (see section 2.5 for details).

#### 3.1.2 Synthesis of PNNP

**Fig. 2:**
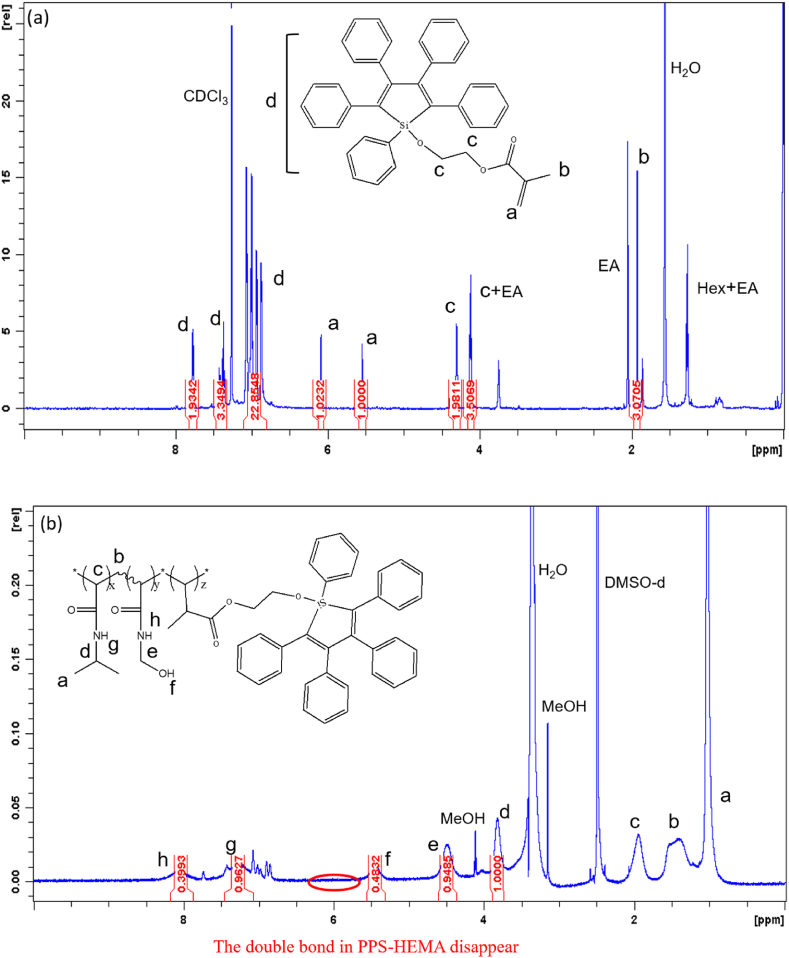
NMR spectra of (a) PPS-HEMA (b) PNNP.

Random PNNP copolymers functionalized with AIE moieties were prepared in a two-step synthesis procedure. In the first step, PPS-HEMA was synthesized. The random copolymer was synthesized via free radical polymerization reaction with PPS-HEMA, NIPAAm and NMA as monomers and AIBN as an initiator; the mixed solution was reacted at 65 °C for 1 day and then subjected to reprecipitation for purification. The structures of PPS-HEMA and random copolymer PNNP were inspected by NMR (Fig. 2). The chemical shift of the double bond in PPS-HEMA (Fig. 2(a), δ: 5.4 and 5.9 ppm) disappeared after polymerization, indicating the reaction was successful. In addition, the proton peak areas in Fig. 2(b) (peaks “e” and “d”) were used to calculate the ratio of monomers (NMA and NIPAAm). The ratio of peak “d” (NIPAAm) to peak “e” (NMA) in the PNNP NMR spectrum was 1.00:0.95. The weight percentage of PPS-HEMA was determined according to the UV spectrum (Fig. S2), and the results showed that PNNP contained 9.16 wt% PPS-HEMA.

### 3.2 Morphology of electrospun nanofibers

Up to this point, we had successfully synthesized two series of random copolymers functionalized with either pyrene or PPS-HEMA moieties through free radical polymerization. Regarding the application of these materials as solid-state fluorescent sensors, a nanofiber film is expected to be preferable to a spin coating thin film due to the high surface-to-volume ratio of the nanofiber. This property maximizes contact between the fluorescent monomer and quenching molecule (i.e., picric acid), which increases the sensitivity of the sensor. Random copolymer nanofibers were fabricated through an electrospinning technique in which BTEAC was used to improve the electrical conductivity of the copolymer solution (see section 2.3 for details). Within the copolymers, the hydrophilic nature of NIPAAm causes the material to swell in aqueous solution, allowing the fluorescent monomers to come into more frequent contact with picric acid in the solution. In order to prevent the dissolution of the nanofibers in aqueous solution due to swelling, we introduced NMA in the copolymers. The hydroxyl groups in NMA moieties crosslink to each other when heated (Fig. S3), which prevents the nanofibers from dissolving in the solvent system and makes the nanofibers more suitable for detection of picric acid in the solvent.

**Fig. 3:**
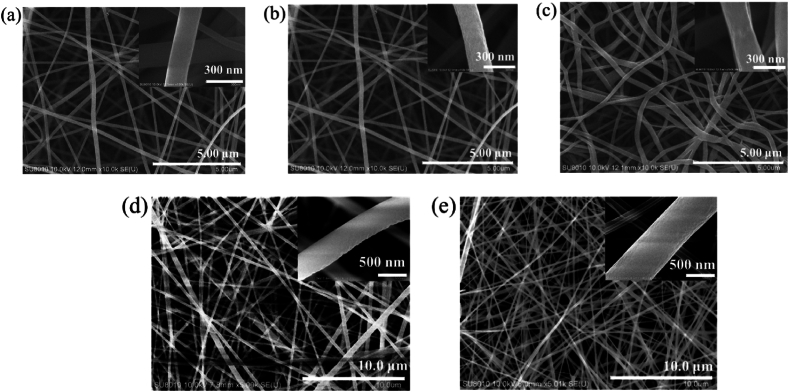
Morphology of PNNS nanofiber (a) before and (b) after cross-linking, and (c) after cross-linking and immersion in water. Morphology of PNNP nanofiber (d) before and (e) after cross-linking.

The morphologies of spun PNNS and PNNP nanofibers are shown in Fig. 3. The nanofibers were found to be smooth and without porous structures or beads. The measured average diameter of PNNS nanofibers was 179 ± 28 nm (Fig. 3(a)), and the diameter of PNNP nanofibers was 235 ± 143 nm (Fig. 3(d)). After cross-linking of the nanofibers at 110 °C for 1–2 days, the morphology (Fig. 3(b) and (e)) of the nanofibers was not observably changed; the diameters of cross-linked PNNS and PNNP nanofibers were 182 ± 29 nm and 255 ± 49 nm, respectively. The results in Fig. 3(c) show cross-linked nanofibers (PNNS) that were immersed into water and allowed to swell for 1 h had an average diameter of 240 ± 28 nm. Importantly, the nanofibers did not dissolve in water, presumably due to the cross-linked NMA moiety. Thus, our data demonstrated that the nanofibers can be immersed in aqueous solution for detection of picric acid.

### 3.3 Fluorescence properties of polymer electrospun nanofibers

#### 3.3.1 Picric acid detection

##### 3.3.1.1 PNNS-based sensors

**Fig. 4:**
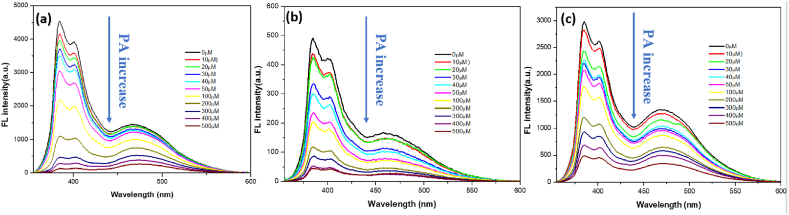
Fluorescence spectra of PNNS exposed to different concentrations of picric acid. PNNS was prepared as a (a) solution, (b) nanofiber and (c) spin coating film.

To test whether our copolymers could be used to detect picric acid, three different forms of the copolymer (aqueous solution, electrospun nanofiber film, and spin coating film) were immersed or mixed with solutions containing different concentrations of picric acid. The fluorescence intensity change for each detection material is shown in Fig. 4(a–c). In the PNNS fluorescence spectrum, two emission bands can be observed, one at 384 nm (monomer band) and the other at 470 nm (excimer band). This pattern is caused by the formation of an excimer in which two pyrene molecules are combined; one is in the ground state, and the other is in the excited state. In general, the emission band of an excimer will be observed at a longer wavelength than that of the monomer band.

**Fig. 5:**
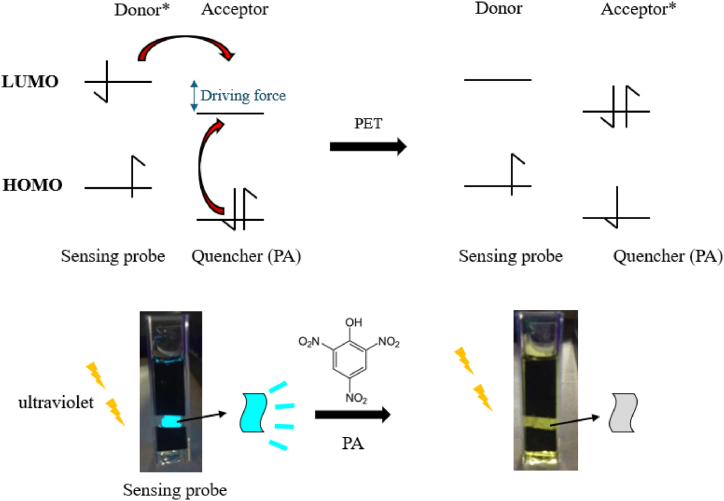
Illustration of the PET mechanism.

In the PNNS detection experiments, picric acid molecules interacted with excited pyrene moieties, and electron transfer occurred from electron-rich pyrene to the electron-deficient picric acid. Thus, the pyrene moieties retained no electron that could return to the ground state, and fluorescence quenching occurred due to this PET mechanism [7,[30], [31], [32]]; the process is illustrated in Fig. 5.

**Fig. 6:**
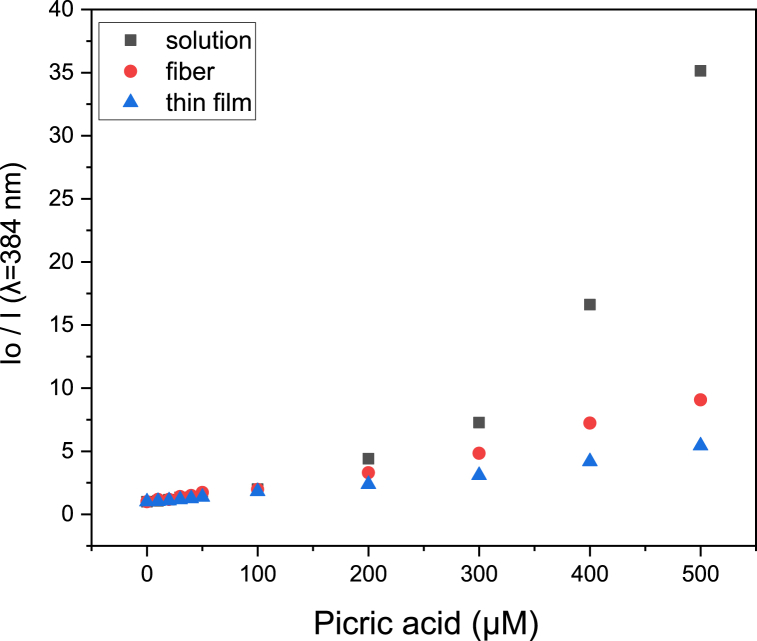
Stern-Volmer plot for PNNS sensors in different forms. Fluorescence intensity of PNNS was recorded at an emission wavelength of 384 nm (λ = 384 nm) with an excitation wavelength of 344 nm.

The data in Fig. 4 show that fluorescence decreases when the picric acid concentration increases for all tested types of random copolymer. To further quantify the sensitivities of each different forms, a Stern-Volmer plot was generated (equation (1) is the Sern-Volmer equation). In a Stern-Volmer plot, the x-axis is the concentration of picric acid solution, and the y-axis is I_0_/I, where I_0_ represents the fluorescent intensity of the copolymer without picric acid, and I represents the fluorescent intensity of the copolymer exposed to picric acid solutions at different concentrations. The slope of the curve is the Stern-Volmer constant (K_sv_), and higher K_sv_ values indicate more sensitivity for the sensor. The Stern-Volmer plot is shown in Fig. 6, and the K_sv_ values derived from each dataset are shown in Table 2.

(1) $$I_{0}/I=1+K_{\text{sv}}\left[PA\right]$$

**Table 2:** K_sv_ (μΜ^−1^) values for PNNS sensors.

PNNS	Solution	Nanofiber	Spin coating film
Picric acid concentration (0–300 μΜ)	0.023	0.012	0.007
Picric acid concentration (300–500 μΜ)	0.133	0.021	0.011

When comparing the sensitivities of solution, nanofiber and thin film states, we found that the solution type of PNNS sensor was more sensitive than the electrospinning nanofiber film. This result is expected because fluorescence quenching requires an interaction between picric acid molecules and fluorescent monomers. In solution, the fluorescent monomers and picric acid molecules are free to intermingle, and the probability of collision is high. However, in solid states like the nanofiber film, the fluorescent monomers are fixed in the substrate, and the picric acid molecules must diffuse into the substrate to interact. Therefore, the probability of collision is lower than in solution. Nevertheless, the higher surface-to-volume ratio of the nanofiber sensor rendered it more sensitive than the spin-coating thin film.

Despite the major advantages of solid sensors, such as reusability and portability, low sensitivity is not very great, this solid sensor is not a perfect sensor, so how to maximize sensitivity in the development of solid sensors. For this purpose, we utilized another type of fluorescent monomer, which has the AIE property. In contrast to the ACQ property (pyrene monomer, Fig. S4), a monomer with AIE will exhibit increased fluorescence intensity when the monomer aggregates. Since the solid state sensor provides an aggregation environment for the monomer, we expected the AIE property may increase the fluorescence intensity of solid-state sensors and provide a larger dynamic range for fluorescence quenching by picric acid.

##### 3.3.1.2 PNNP-based sensors

**Fig. 7:**
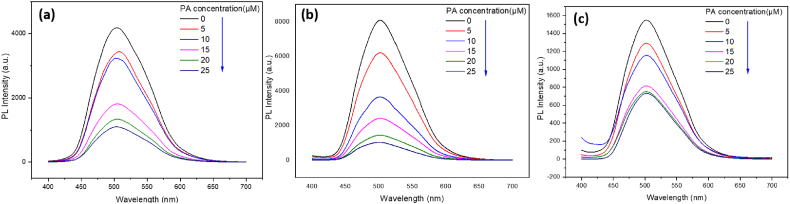
Fluorescence spectra of PNNP exposed to different concentrations of picric acid. PNNP was prepared as a (a) solution, (b) nanofiber and (c) spin coating film.

**Fig. 8:**
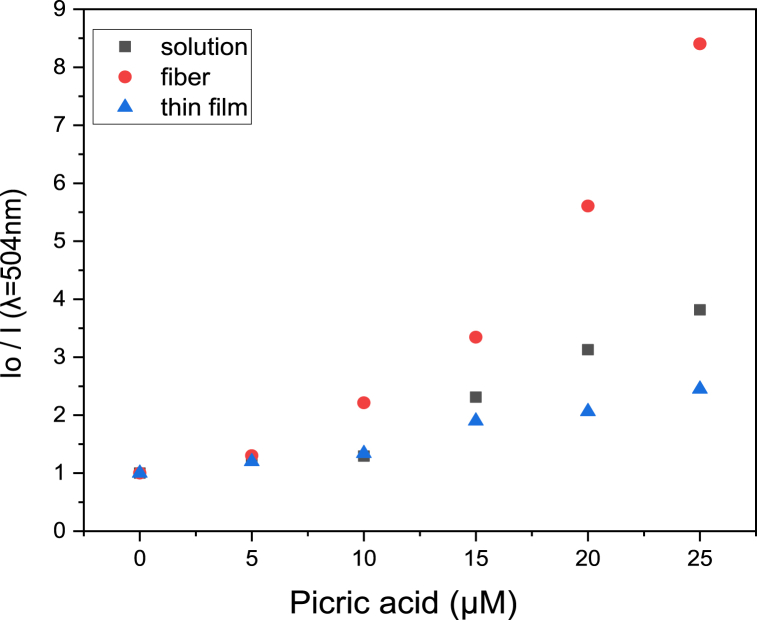
Stern-Volmer plot for PNNS sensors. The fluorescence intensity of PNNP was recorded at an emission wavelength of 504 nm (λ = 504 nm) and an excitation wavelength of 376 nm.

Fig. 7(a–c) shows the results of picric acid detection with PNNP sensors, and we can see from the graphs that the fluorescence intensity decreases when the picric acid concentration increases for each tested type of sensor. The quenching mechanism is the same as for the PNNS sensor (for detailed HOMO and LUMO information, see Fig. S5). An excited electron in the electron-rich PPS-HEMA will be transferred to picric acid [7]. The electron-deficient picric acid will create a driving force for electron transfer between PNNP and picric acid. The Stern-Volmer plot is shown in Fig. 8, and the K_sv_ values are shown in Table 3.

**Table 3:** K_sv_ (μΜ^−1^) values for PNNP sensors.

PNNP	Solution	Nanofiber	Spin coating film
Picric acid concentration (0–25 μΜ)	0.119	0.276	0.059

Based on the results shown in Fig. 8 and Table 3, we observed that the nanofiber sensor is more sensitive than the solution type. This improvement in sensing ability can be attributed to the AIE property of PPS-HEMA (Fig. S6). In the random copolymer, the motion of the fluorescent monomer will be restricted by the polymer chain, so the ability to undergo aggregation will be higher in the solid state. While aggregation causes fluorescence quenching for PNNS, it enhances PNNP fluorescence, which results in a higher signal before picric acid detection. This higher baseline signal contributes to better sensitivity for PNNP-based probes due to the larger dynamic range for fluorescence quenching by picric acid.

We next compared the sensitivities of our sensors with those reported in other recent studies on sensors for picric acid detection (Table 4). The sensitivity of PNNS-based sensors was not as high as previously developed sensors. It seems that the ACQ property of pyrene restricts its utility as a sensor. The AIE monomer showed good sensitivity in comparison to other sensors. Notably, when PNNP was prepared as a nanofiber, the sensing ability was greater than previously developed sensors, presumably due to the increased aggregation and high surface-to-volume ratio in this solid sensor probe.

**Table 4:** Comparative information on published sensors.

Material	Fluorescence property	Sensor type	K_sv_ (μM^−1^)	Ref.
TAA-based probe	AIE	solution	0.117	[33]
p-PBP	ACQ	solution	0.077	[34]
DNDPE-conjugated polymer	AIE	solution	0.0470	[35]

### 3.4 Reversibility of nanofibers

**Fig. 9:**
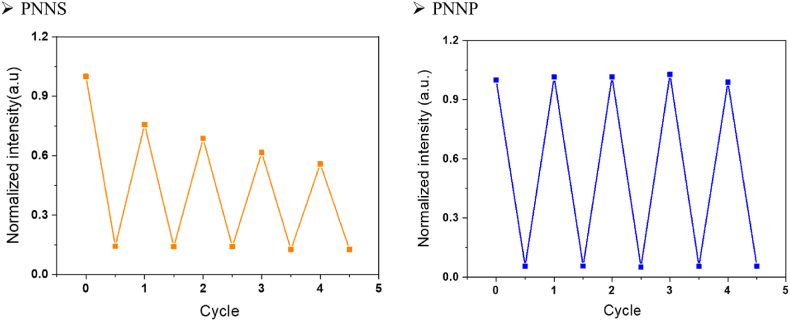
Reversibility of (a) PNNS (b) PNNP nanofiber sensors.

In contrast to solution-type sensors, solid-type sensors possess a major potential advantage of reversibility. To test the reversibility of our sensors, the nanofiber films were fixed on quartz slides and inserted into a quartz cell without or with picric acid solution for measurement. Afterward, the nanofiber film was rinsed with methanol or deionized water, and subsequent measurements of fluorescence intensity were made. This process was repeated several times to verify the reversibility of the nanofiber sensors (Fig. 9). After the sensor was quenched in picric acid solution, pure methanol (for PNNS, Fig. 9(a)) or deionized water (for PNNP, Fig. 9(b)) was used to clean the sensor. Upon cleaning, both sensors recovered high fluorescence intensity. Since the signal could be recovered to a level similar to the original intensity, and the fluorescence decrease upon picric acid exposure was similar for every cycle, we concluded that the nanofiber sensors appear to be reusable for detecting picric acid.

## 4 Conclusion

In this study, we successfully fabricated electrospun nanofiber films from PNNS and PNNP fluorescent copolymers, and we evaluated the performance of these sensors for picric acid detection. To characterize the nanofibers, morphology was observed by FE-SEM, which revealed that the fiber diameters did not change significantly after cross-linking. From our picric acid detection experiments, we found that both random copolymers can be used to detect the picric acid through PET-mediated quenching behavior. However, the PNNP sensor has higher sensitivity than PNNS, presumably due to its AIE property. Finally, we showed that both nanofiber films can be reused after washing with pure methanol or deionized water.

## CRediT authorship contribution statement

**Hong-Jia Lin:** Writing – review & editing, Writing – original draft, Methodology, Formal analysis, Data curation, Conceptualization. **Po-Hsiang Tseng:** Writing – review & editing, Writing – original draft, Formal analysis, Data curation, Conceptualization. **Wen-Chung Wu:** Writing – review & editing, Writing – original draft, Visualization, Validation, Supervision, Resources, Project administration, Investigation, Funding acquisition.

## Data availability statement

All data are included in this article and supplementary material.

## Declaration of competing interest

The authors declare that they have no known competing financial interests or personal relationships that could have appeared to influence the work reported in this paper.
