# Supplementary material for: Fluorescent sensing copolymers: Synthesis, nanofiber fabrication and application in picric acid sensors
Source: Heliyon. 2024 Nov 29;10(23):e40786. doi: 10.1016/j.heliyon.2024.e40786 (PMC11652853; doi:10.1016/j.heliyon.2024.e40786)
Supplement: Multimedia component 1 [file mmc1.docx]

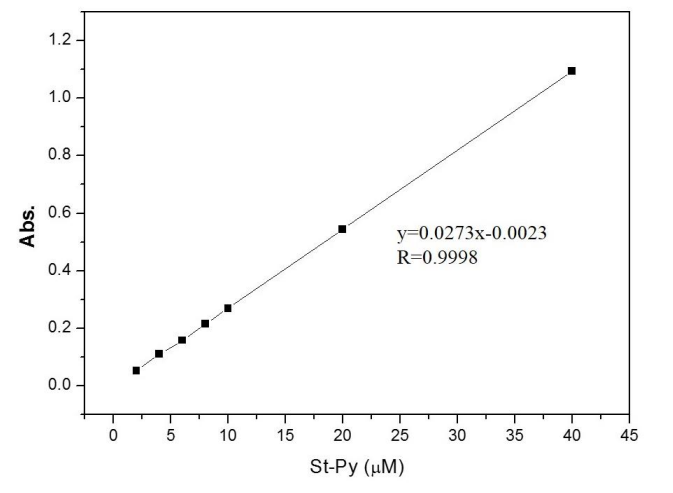


Figure S1. The calibration curve of St-Py in MeOH. The absorption value was recorded at the wavelength of 344 nm.


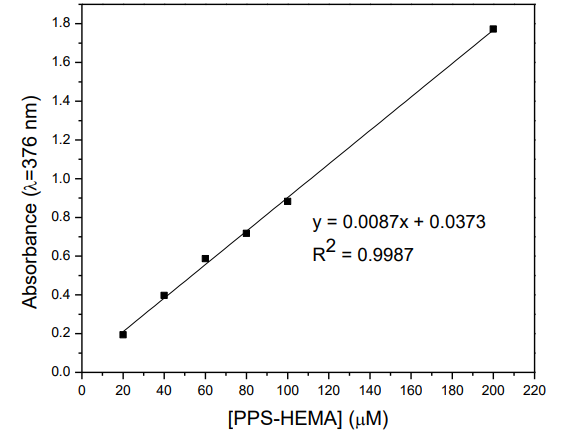


Figure S2. The calibration curve of PPS-HEMA in MeOH. The absorption value was recorded at the wavelength of 376 nm.


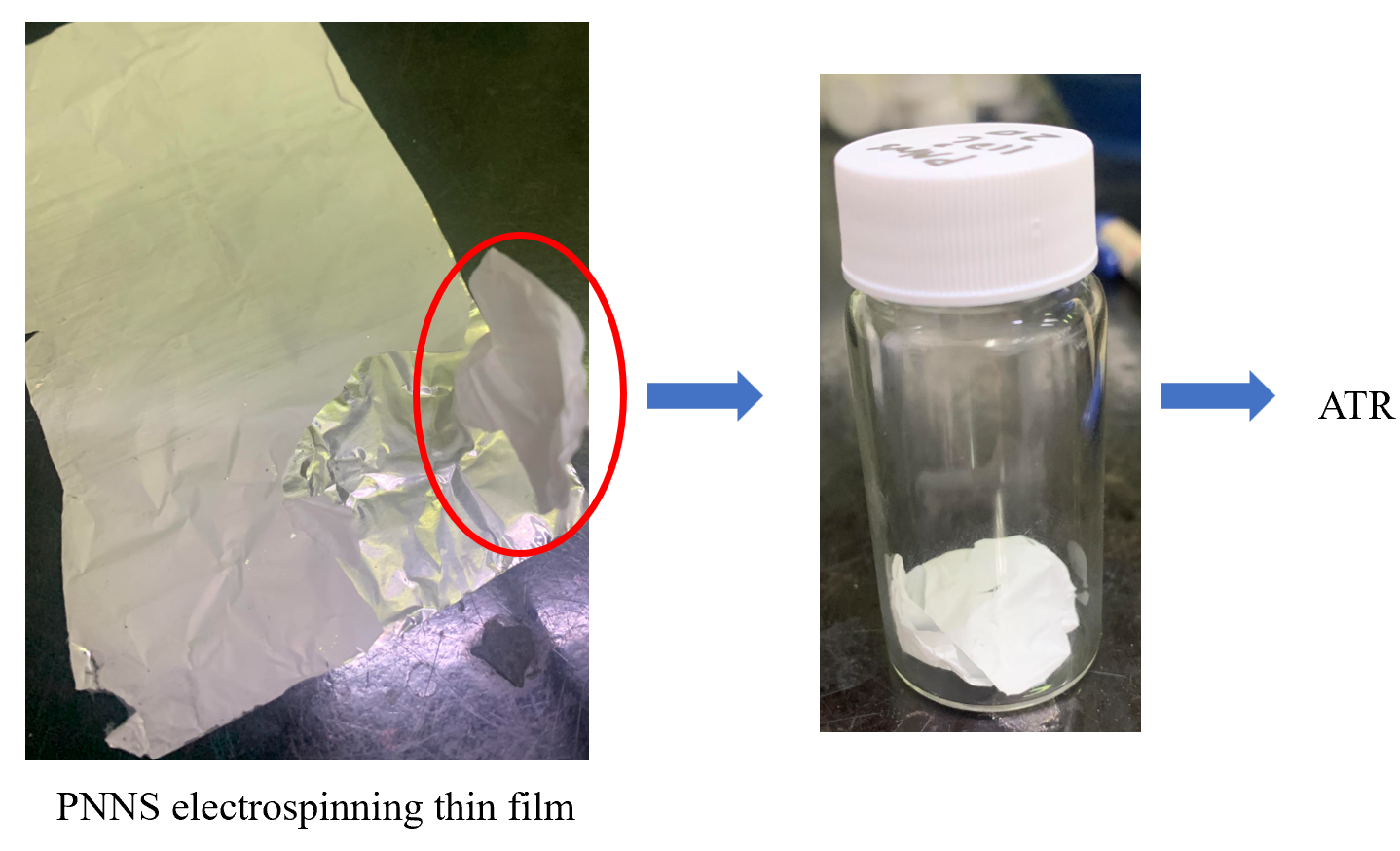


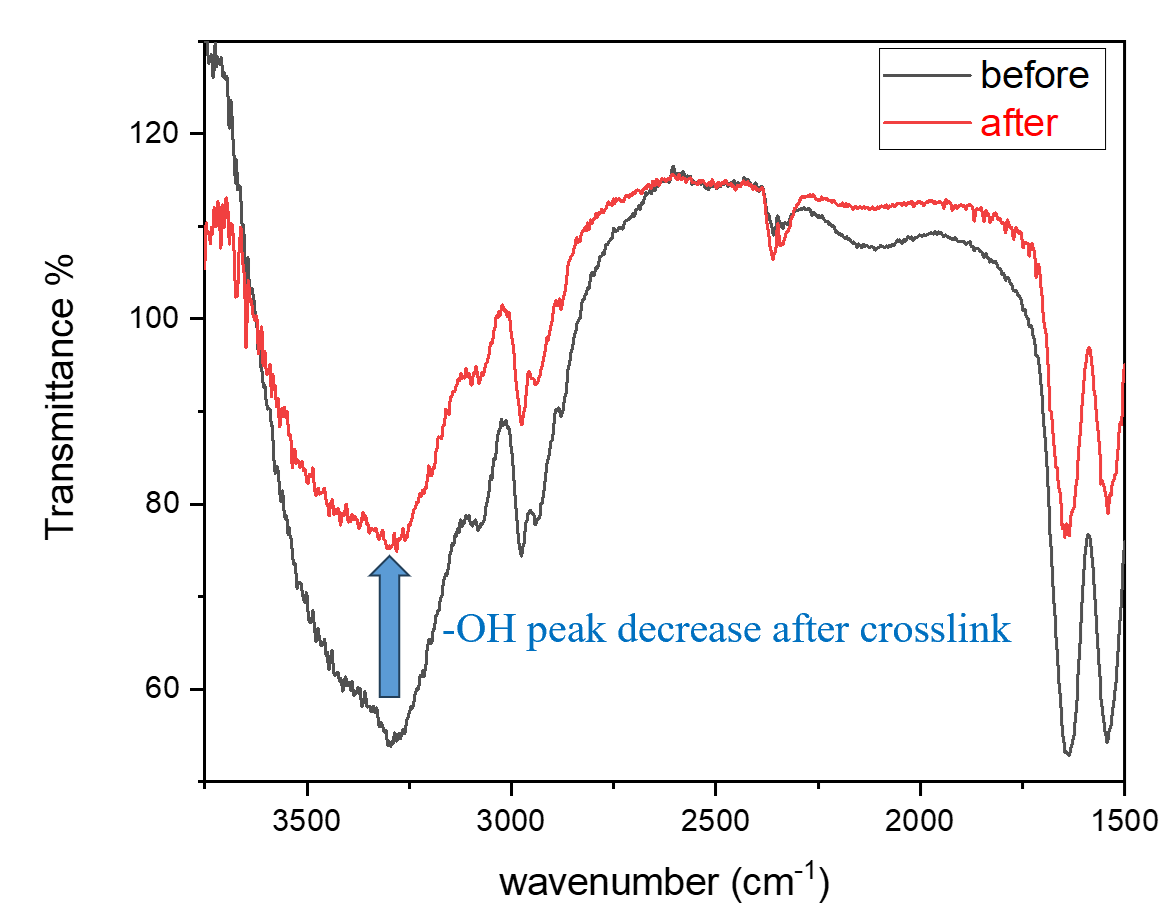


Figure S3. The image of electrospinning thin film, the crosslinking process was conducted under 110 ^o^C for 2 days. Then, a slice of thin film was torn from the aluminum sheet and detected by ATR. The result shows the decreasing peak at 3300 (cm^-1^), meaning that the hydroxyl group was decreased during the crosslinking process.

Figure S4. The result of PNNS fluorescence intensity at different temperatures in water/methanol (1/1, v/v) system. When temperature increase, the property of NIPAAm in PNNS will change from hydrophilic into hydrophobic, it means that PNNS tend to aggregate in this solvent system. The fluorescence intensity decreases when temperature arise show that the PNNS polymer own ACQ property.


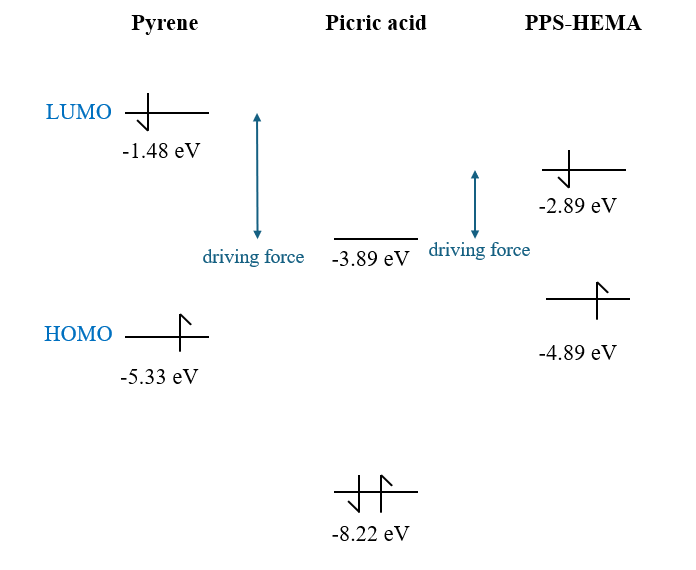


Figure S5, the orbital of pyrene, PPS-HEMA and picric acid. The HOMO and LUMO information of pyrene and picric acid were according to the reference: *Xiangcheng Sun, Yixin Liu, George Shaw, Andrew Carrier, Swayandipta Dey, Jing Zhao, and Yu Lei, Fundamental Study of Electrospun Pyrene−Polyethersulfone Nanofibers Using Mixed Solvents for Sensitive and Selective Explosives Detection in Aqueous Solution, 7 (2015) 13189-13197.* While the molecular orbital of PPS-HEMA (for PNNP) was calculated by “Materials studio DMoL3, density functional theory”.


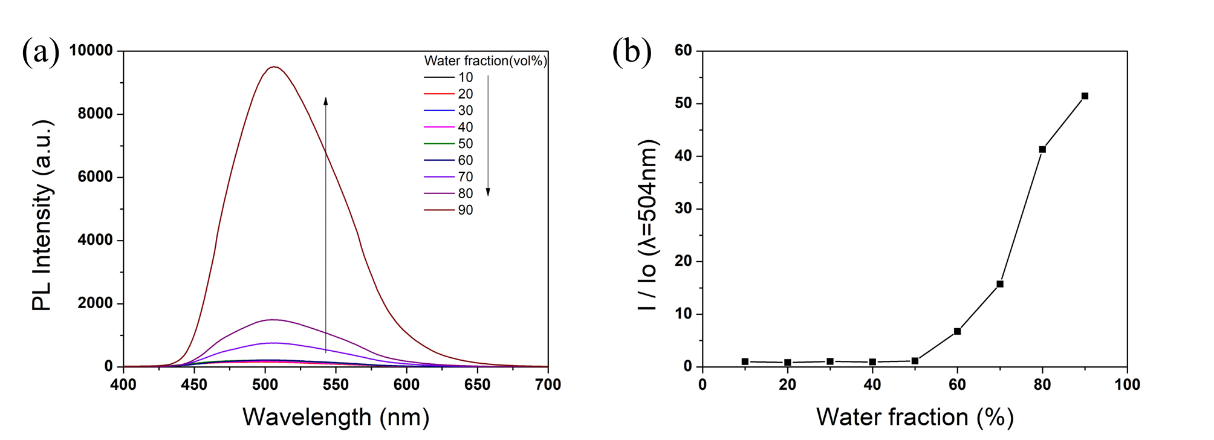


Figure S6. (a) the fluorescence intensity of PPS-HEMA (1mM) in different compositions of solution (THF/water) (b) the fluorescence intensity of PPS-HEMA recorded at the wavelength of 504 nm in different compositions of solution (THF/water).

When the water percentage increased, the PPS-HEMA tended to aggregate. The results showed that the signal intensity increased when the water percentage increased, meaning that the rise in signal intensity was related to the aggregation of PPS-HEMA.
